# Bilateral central pain sensitization in rats following a unilateral thalamic lesion may be treated with high doses of ketamine

**DOI:** 10.1186/1746-6148-9-59

**Published:** 2013-03-27

**Authors:** Aude Castel, Pierre Hélie, Francis Beaudry, Pascal Vachon

**Affiliations:** 1 Faculty of Veterinary Medicine, Departments of Veterinary Biomedicine, Saint-Hyacinthe, Quebec, Canada; 2 Pathology & Microbiology, University of Montreal, Saint-Hyacinthe, Quebec, Canada

**Keywords:** Central pain, Thalamus, Hematoma, Ketamine, Allodynia, Hyperalgesia

## Abstract

**Background:**

Central post-stroke pain is a neuropathic pain condition caused by a vascular lesion, of either ischemic or hemorrhagic origin, in the central nervous system and more precisely involving the spinothalamocortical pathway responsible for the transmission of painful sensations. Few animal models have been developed to study this problem. The objectives of this study were to evaluate different modalities of pain in a central neuropathic pain rat model and to assess the effects of ketamine administered at different doses. Animals were evaluated on the rotarod, Hargreaves, Von Frey and acetone tests. A very small hemorrhage was created by injecting a collagenase solution in the right ventral posterolateral thalamic nucleus. Following the establishment of the neuropathy, ketamine was evaluated as a therapeutic drug for this condition.

**Results:**

Histopathological observations showed a well localized lesion with neuronal necrosis and astrocytosis following the collagenase injection that was localized within the VPL. No significant change in motor coordination was observed following surgery in either the saline or collagensae groups. In the collagenase group, a significant decrease in mechanical allodynia threshold was observed. A sporadic and transient cold allodynia was also noted. No thermal hyperalgesia was seen following the collagenase injection. Ketamine was then tested as a potential therapeutic drug. A significant decrease in motor coordination was seen only following the administration of 25 mg/kg of ketamine in both groups. An alleviation of mechanical allodynia was achieved only with the high ketamine dose. The minimal effective ketamine serum concentration (150 ng/mL) was only achieved in animals that received 25 mg/kg.

**Conclusions:**

An intrathalamic hemorrhage induced a bilateral mechanical allodynia in rats. Cold hyperalgesia was observed in 60% of these animals. Mechanical allodynia was alleviated with high doses of ketamine which corresponded with therapeutic plasmatic concentrations.

## Background

Central post stroke pain (CPSP) can develop following a vascular lesion of cortical and subcortical structures [1]. CPSP was first associated with thalamic infarcts by two French neurologists [2] and they termed this condition « thalamic syndrome ». However, further investigations suggested that central pain could be induced by stroke in other structures all along the spinothalamic pathway [3]. Development of pain in stroke patients is of great concern since it deteriorates their quality of life making daily activities difficult, compromising rehabilitation efforts, altering mobility, concentration, mood [4,5] and eventually leading to depression [4,6]. Little is known about the treatment of this condition and therefore CPSP management represents a real medical challenge and requires further understanding of its mechanisms.

CPSP is characterized by spontaneous and evoked pain [7,8]. Allodynia and thermal hyperalgesia, can be typical clinical signs recognized in patients and they are induced by thermal (especially cold) and mechanical stimuli [7,9]. In recent population-based studies, CPSP incidence between 7.3% and 10.5% is reported amongst stroke patients [10,11]. Some studies suggest that the prevalence of CPSP depends on the location of the lesion and it appears to develop more commonly after medullar infarctions or thalamic lesions [12-14]. A higher incidence occurs following a hemorrhage in the posterolateral and dorsal thalamic nuclei (32% and 25% respectively) [12]. Additionally, it seems that right sided lesions predominate in CPSP patients at both cortical and thalamic levels [15]. Functional imaging studies have helped us to identify structures involved in CPSP [16-18] and ventroposterior (VP) thalamic nuclei play a key role in this condition [19,20].

Many mechanisms have been proposed to explain central neuropathic pain [21-23]. In central pain, nociceptive neuronal hyperactivity suggests a central sensitization mechanism possibly involving N-methyl-D-aspartate (NMDA) receptors [24,25]. Ketamine, a well-know NMDA receptor antagonist, has been used to treat various neuropathic pain disorders [26-29]. Furthermore, the use of ketamine to treat CPSP has been shown efficacious in CPSP patients [30,31]. Therefore NMDA receptors could play an important role in CPSP.

The use of animal models can help us understand the mechanisms involved in CPSP and allow testing of new therapeutic approaches for this condition. Wasserman and Koeberle [32] were able to recreate central pain by injecting collagenase within the ventroposterolateral thalamus nucleus of rats creating an intracerebral hemorrhage. The objectives of the present study were to evaluate if central pain persists over a longer period of time, using Wasserman and Koeberle’s model, and to evaluate in a preliminary study if ketamine could be used for the alleviation of pain in this model.

## Results

Following surgery, some rats (40-50%) in the collagenase group were reluctant to be manually held and vocalized during manipulations. These occurrences were never observed prior to the surgery, neither were they observed in our previous experiments using a peripheral neuropathy rat model.

### Evaluation of the central pain model

#### Motor coordination evaluated with the rotarod test

**Figure 1:**
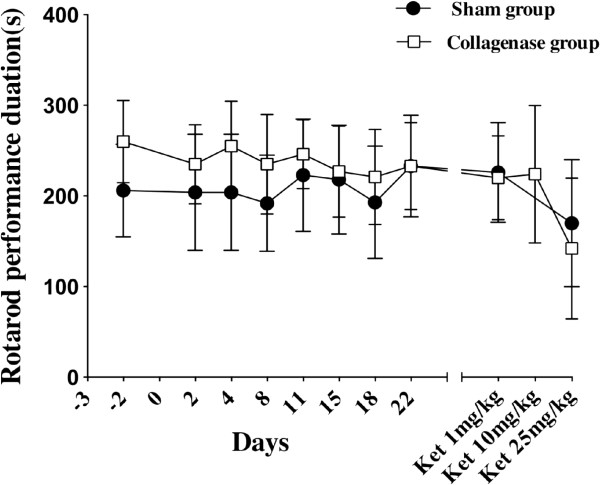
**Motor coordination evaluated with the rotarod test in Spraque-Dawley rats.** Animals received stereotaxically either saline (n = 8) or a collagenase solution (n = 7) in the ventroposterolateral nucleus of the thalamus. No significant difference was seen between sham and collagenase groups at baseline and at different post surgical time points up to 22 days (F_1,13_ = 0.18, ns). Only the IP administration of 25 mg/kg of ketamine caused a significant impairment (p < 0.05) in motor coordination seen in both groups.

No significant difference between sham and collagenase groups was observed on the rotarod test (F_1,13_ = 0.18, ns). Results are shown in Figure 1.

#### Heat hyperalgesia evaluated with Hargreave’s test

**Figure 2:**
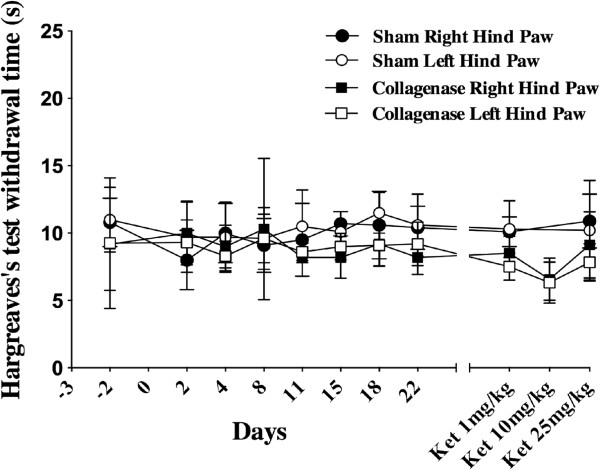
**Heat sensitivity evaluated with Hargreave’s test in Spraque-Dawley rats.** Animals received stereotaxically either saline (n = 8) or a collagenase solution (n = 7) in the ventroposterolateral nucleus of the thalamus. No significant difference in the thermal thresholds between both groups was seen at baseline and at different post surgical time points up to 22 days (F_1,13_ = 0.23, ns).

At baseline and during the first part of the experimentation, there was no significant difference between experimental groups for both hind limbs (F_1,13_ = 0.23, ns). Results are shown in Figure 2.

### Mechanical allodynia evaluated with Von Frey filaments

**Figure 3:**
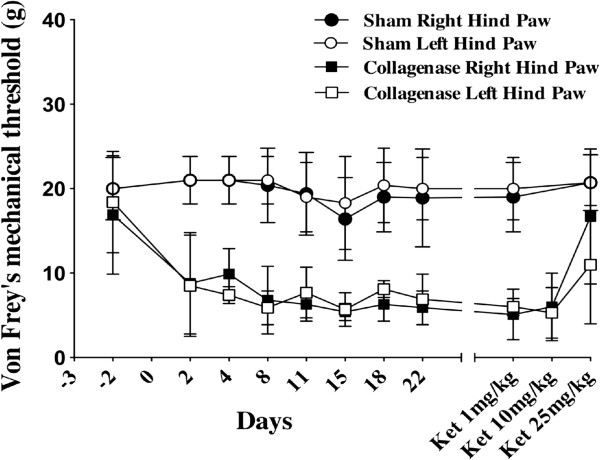
**Evaluation of mechanical allodynia using Von Frey filaments.** No significant difference was seen for both hind limbs at baseline values between both saline and collagenase groups. As early as the second post-surgical day, a significant decrease in the mechanical threshold in both hind limbs was noted for the collagenase group when compared to sham animals in the right (F_1,24.5_ = 130.4, p < 0.0001) and left (F_1,28.1_ = 207.8, p < 0.0001) hind paws. Only the 25 mg/kg of ketamine dose reduced mechanical allodynia in the contralateral (p < 0.0001) and ipsilateral (p < 0.01) hind paws.

Results are presented in Figure 3. Prior to surgery, there was no difference in mechanical sensitivity for both hind paws when comparing sham and collagenase groups. Following surgery, significant differences in mechanical thresholds occurred in the right (F_1,24.5_ = 130.4, p < 0.0001) and left (F_1,28.1_ = 207.8, p < 0.0001) hind paws in the collagenase group. In the sham group, no difference was seen between baseline values and post-surgical results.

#### Evaluation of cold allodynia with the acetone test

**Figure 4:**
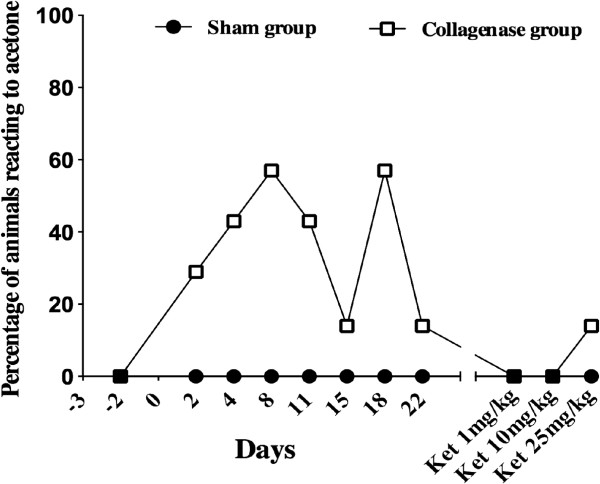
**Percentage of Sprague Dawley rats reacting to acetone to determine cold hyperalgesia.** Although no significant difference was seen when comparing results either prior or after surgery for either sham or collagenase groups, some animals within the collagenase groups clearly appear to be more sensitive to cold.

Animals in the sham group never had reactions to acetone that were different from baseline. These animals either didn't move or lifted their paw rapidly only once at the acetone application. Conversely, an increase in the number and duration of movements in the collagenase group with acetone (graded 2) was seen with peak reaching up to 57% of rats on days 8 and 18 post-surgery. Some of these animals would have marked reactions with either duration being around 10s, or number of movements being as high as 10 (Figure 4). Right and left hind limbs were affected in these animals. However, these results failed to show any statistical significance when compared to the sham group. The percentage of animal in the collagenase group that reacted to acetone slowly decreased and the reactions were comparable to baseline by the end of the study.

### Evaluation of ketamine

Rats from the sham and the collagenase groups received a single IP injection of ketamine (100 mg/mL) of 1, 10 and 25 mg/kg on respectively days 28, 30 and 32 post-surgery. Behavioral tests started 30 min after the injection of the ketamine solution and the experimenter was blinded to the treatment drug and concentrations. Injections of ketamine at 1 and 10 mg/kg did not affect the rotarod performance however with 25 mg/kg of ketamine, it was significantly impaired for animals in the sham (p < 0.03) and collagenase (p < 0.05) groups (Figure 1). The thermal sensitivity did not differ between sham and collagenase groups following the administration of ketamine at 1, 10 and 25 mg/kg (Figure 2). Values were comparable to baseline suggesting that even the high dose of ketamine did not affect reflex activity. Ketamine had an alleviating effect on mechanical allodynia (Figure 3). With 1 and 10 mg/kg, there was no effect on mechanical sensitivity in either hind paw however with 25 mg/kg of ketamine mechanical allodynia was reduced in the contralateral (p < 0.0001) and ipsilateral (p < 0.01) hind paws.

### Plasmatic ketamine concentrations

At the end of the study (day 35), 6 rats (3/dose) from the sham group were used to evaluate the plasmatic concentration of ketamine following an IP administration of 10 or 25 mg/kg. Following 10 mg/kg ketamine IP, mean plasmatic concentrations at 30 min, 1 and 2 h post administration were respectively 78 ± 23, 48 ± 14 and 22 ± 8 ng/mL. Following 25 mg/kg ketamine IP mean plasmatic concentrations at 30 min, 1 and 2 h post administration were respectively 142 ± 47, 96 ± 75 and 59 ± 25 ng/mL. Maximal concentrations following 25 mg/kg seen at 30 min and 1 h post-ketamine administration range from 181 to 204 ng/mL.

### Histopathological evaluation

**Figure 5:**
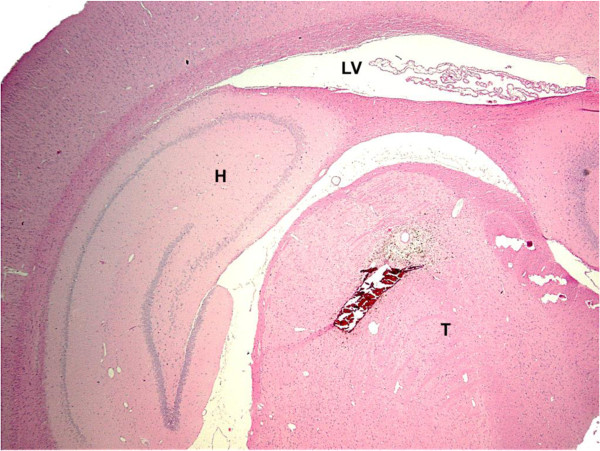
**Localisation and size of the collagenase lesion.** Photomicrograph of a tranverse rat brain section (4 μm) showing a well circumscribed lesion injection in VPL nucleus of the thalamus following a collagenase solution (H&E stain, x12.5). H : hippocampus, LV : lateral ventricle, T : thalamus.

**Figure 6:**
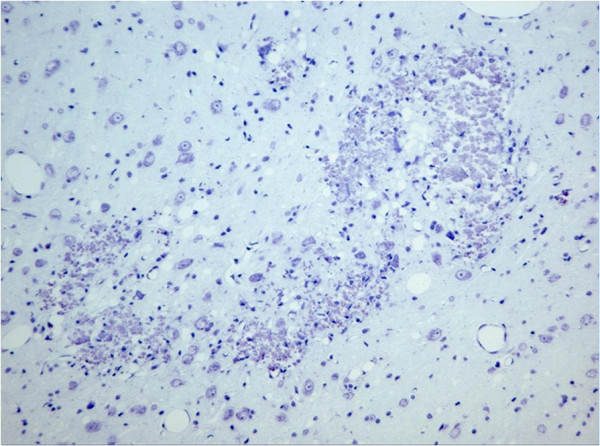
**Photomicrograph of the collagenase lesion in VPL nucleus of the thalamus stained with cresyl violet.** A marked decrease in the number of normal neurons is appreciated as well as area of neuronal degeneration in the area of the VPL. (x100).

**Figure 7:**
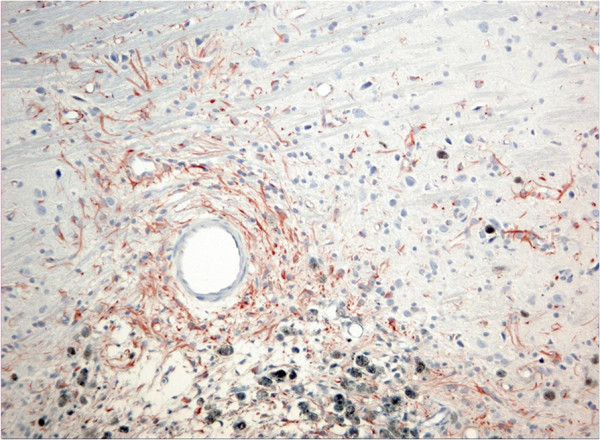
**Photomicrograph of the collagenase lesion after GFAP immunohistochemistry in a brain section following a collagenase solution injection in VPL nucleus of the thalamus.** Presence of astrocytosis (filamentous structures) is visible in the lesioned area. Mineralization (dark blue granular deposits) can be observed surrounding the lesion (lower part of the photomicrograph). (x60).

Microscopic evaluation of brain slices from the sham group showed no necrotic neuron and no astrocytosis in the VPL nucleus. In the collagenase group, small well circumscribed lesions were observed in the lateral thalamic nuclei (including the VPL) (Figure 5). On H&E sections, neuronal, and in some cases neuropil degeneration was observed and a large number of small nuclei was present in the lesioned areas, most probably reactive astrocytes. A marked decrease in the number of normal neurons is appreciated with the cresyl violet stain (Figure 6). Reactive astrocytes (filamentous structures) are clearly seen with GFAP immunochemistry at the site of the lesion (Figure 7). With GFAP immunohistochemistry, some mineralization (dark blue granular clumps) was observed in the vicinity of the lesion. The contralateral side of the corresponding thalamic lesioned area was uneventful.

## Discussion

In this study, we used a model of central pain in rats induced by an intrathalamic hemorrhage in the ventroposterolateral nucleus [32] to evaluate the development and maintenance of neuropathic pain and assess ketamine as a potential therapeutic drug. We observed a significant mechanical allodynia up to 30 days after the intracerebral lesion, and a transient cold allodynia in up to 60% of these animals. No heat hyperalgesia occurred in the lesioned animals. These results reflect clinical signs such as mechanical allodynia and cold hyperalgesia seen in human with post-stroke pain [7,9]. We also found that only the highest dose of ketamine administered (25 mg/kg IP) was able to alleviate the mechanical allodynia. However since animal were also affected on the rotarod, a decrease of motor control could be a confounding variable.

Mechanical and cold allodynia, have been described in patients with thalamic stroke [1,8,9,11,33]. In one particular study among CPSP patients, allodynia was relatively common with a higher incidence in patients with thalamic lesions [1]. Tactile hypoesthesia may also occur in the absence of tactile allodynia whereas normal tactile detection threshold can be associated with the presence of tactile allodynia [33]. In our experiment, a decrease in the mechanical allodynia threshold in both hind paws appeared following surgery and persisted over time. These results are concordant with those of Wasserman and Koeberle [32] who observed mechanical allodynia for 7 days following the collagenase injections. Mechanical allodynia has also been reported following kainate lesions in the VPL, however, animals were not tested beyond 48 h [34]. Wasserman and Koeberle [32] reported that mechanical allodynia was unilateral and contralateral to the side of the lesion which could be associated with some methodological differences as baseline evaluations were not performed and the mechanical sensitivity was evaluated for a short period.

Change in thermal sensitivity, either to cold and less often to hot temperatures, is commonly found in CPSP patients [8,33]. In our study we didn’t observe a decrease in heat threshold following the intracerebral hemorrhage. La Buda et al. [34] and Wasserman and Koeberle [32] noted a heat sensitization for 2 and up to 21 days, respectively. Wasserman and Koeberle [32] used the hot plate test which could give different results from our findings with Hargreave’s test. We have no clear explanation for these difference results at the present time.

Abnormal sensitivity to cold has been described in CPSP patients [8,9,11,33]. The prevalence of cold allodynia varies between 18 and 66% in CPSP patients with thalamic lesions [1,11,33]. Conversely, one study reports that CPSP patient suffered mainly from cold hypoesthesia [33]. Our results reflect findings in human patients as cold allodynia appeared as a sporadic sensory abnormality [1,11].

Bilateral sensitization to thermal and mechanical stimuli has been reported in rodent following unilateral electrolytic or demyelinating lesions in the spinothalamic tract and following a hematoma targeted at the basal ganglia, extending into thalamic nuclei [23,35]. In a mouse model of left middle cerebral artery ischemic stroke, hyperesthesia developed in both hind paws and mechanical allodynia was present only in the ipsilateral side of the lesion [36]. Lesion of lateral thalamic neurons or projection fibers to the intralaminar thalamic nuclei could explain our findings since disinhibition in these nuclei may occur with VPL lesions [37]. Nociceptive intralaminar thalamic neurons have very large often bilateral receptive fields and if sensitized could account for the bilateral signs observed in our study [38]. In rats, there are also bilateral projections to the thalamus from spinothalamic neurons which may explain bilateral signs [39,40]. In human, bilateral sensory abnormalities have been described in a few cases [41]. Bilateral hypoesthesia to cold and warm has been reported in CPSP patients [33]. Bilateral processing of pain hypersensitivity has been shown to occur in human and can be explained by changes involving the putamen, thalamus, insula, anterior cingulate cortex, and secondary somatosensory cortex, which may all participate in the bilateral spread of pathological pain resulting from unilateral injury [42-44]. All these proposals may explain bilateral pain following a unilateral lesion of spinothalamic structures however they are speculative and need to be addressed in future experiments.

The neuropathy onset following collagenase injection was rapid in our animal model. In humans, few patients develop central pain immediately after the stroke and it occurs in the majority of patients within 1 to 3 month of the stroke [8,9]. In only one retrospective analysis of 175 patients with thalamic hemorrhage, the delay to develop thalamic syndrome was between 3 and 15 days after the lesion [12]. We have no clear explanation for the difference between findings in the animals and clinical findings in human. One hypothesis would be that plasticity and neuronal reorganization are faster in rodent’s nervous system than in humans. Although, we have not performed chronic evaluations (at least 3 months), persistence of mechanical allodynia was well established in our study which leads us to suspect that this model is suitable to study chronic central pain syndrome mechanisms.

In humans, ketamine can alleviate allodynia and hyperalgesia associated with chronic neuropathic pain state of either peripheral or central origins [26,30,31]. In a randomized, double blind crossover study with ketamine, continuous and evoked pain (notably mechanical allodynia) in patients suffering central dysesthesia following spinal cord injury, was markedly reduced [28]. Our study shows that relatively high doses of ketamine could reduce mechanical allodynia induced by an intrathalamic hematoma in the thalamus. There were no effects of ketamine for both the saline and collagenase groups on the Hargreave’s test, which suggest that the high dose did not affect reflex activity. With von Frey filaments, it is the sensitivity to the mechanical touch stimulus that is evaluated, the reaction time component of this task is not measured, and we would therefore suggest that ketamine does have an effect of mechanical allodynia.

Evaluation of plasmatic concentration following injection of the different doses in rats revealed that the minimal effective plasma concentration of ketamine (approx. 200 ng/mL) was achieved only in a few animals at 25 mg/kg IP, which is the same plasmatic therapeutic concentration needed for treatment of neuropathic pain in humans [45]. Eide et al. [28] have shown that there was a highly significant correlation between the serum concentration of ketamine and the reduction of continuous pain following a single bolus administration of the drug. However, unique administration could be of short duration if a targeted plasmatic concentration is necessary to treat central pain. Other beneficial effects with chronic administrations could be associated with the modulation of synaptic plasticity over time. Unfortunately, high dose of ketamine impaired motor coordination which would be detrimental in CPSP patients. Reported side effects in human include among others dysphoria, sedation, light-headedness [26,30,33] as well as motor impairment, delirium, amnesia, anxiety, and panic attacks [27]. Long-term use of ketamine for the management of CPSP should be recommended only after weighing the pros and cons with the patients as well as considering first other therapeutic strategies.

Allodynia and hyperalgesia in CPSP patients may result from neuronal hyperexcitability which may be related to central sensitization [25,46]. Since ketamine appears to reduce mechanical allodynia following intrathalamic hemorrhage, NMDA receptors activation may be one of the mechanisms generating CPSP. Blockade of NMDA receptors reduces central pain by decreasing neuronal hyperexcitability [47] and therefore this drug is justified for the treatment of central neuropathic pain. In central pain, damage to neurons from excitatory amino acids (ex. glutamate) related to NMDA receptors over-activation could be a contributing factor to the pathology [46]. The evaluation of ketamine in this model was done at the end of our study and it was only performed as a preliminary study for future investigations.

## Conclusions

A unilateral intracerebral thalamic hemorrhage in rats following injection of a collagenase solution within the right VPL caused bilateral allodynia. A long lasting mechanical allodynia developed soon after the surgery. Some animals also developed a transient cold allodynia. Administration of ketamine, a NMDA receptor antagonist, decreased ipsilateral mechanical allodynia. These results suggest that NMDA receptors may be involved in the sensitization mechanism of central post-stroke pain.

## Methods

### Animals

Sixteen Sprague–Dawley rats (Charles River, St-Constant, QC, Canada) between 7 and 8 weeks of age (BW: 300–350 g) were purchased for this study. They were housed in a standard environment (fresh filtered air: 15 changes/h, temperature: 21 ± 3°C, humidity: 40-60% and light–dark cycle: 12 h:12 h). Prior to the surgery, rats were pair-housed in polycarbonate cages (Ancare, Bellmore, NY, USA) on hardwood bedding (Beta chip, North-Eastern Products Co., Warrenburg, NY, USA) and acclimated to their environment for 7 days prior to the initiation of the study. Following surgery, the animals were housed individually to avoid suture chewing. The animals were fed a rodent chow (Charles River Rodent Chow 5075, St-Constant, Qc) and received tap water, both *ad libitum*. The University of Montreal’s Faculty of Veterinary Medicine Institutional Animal Care and Use Committee approved the experimental protocol prior to animal use in accordance with the guidelines of the Canadian Council on Animal Care.

### Surgical techniques

The surgery was performed according to the model previously described by Rosenberg et al. [48]. Rats were divided in two equal sized groups (sham and collagenase). Under general anesthesia, one group received the collagenase solution (collagenase group) and the other sterile saline (sham group) using a stereotaxic apparatus (David Kopf Instruments, Tujunga, CA, USA). Animals were anesthetized using vaporized isoflurane (5% induction, 3% maintenance) (Aerrane, Baxter, Mississauga, ON, Canada) in oxygen. Animals were placed on a regulated heating blanket and body temperature was monitored using a rectal probe (Thermalert TH-8, Physitemp, Clifton, NJ, USA) to keep temperature within normal limits (36-37°C). Pulse oximetry (CANL-425 V, Med Associates, St-Alban, VT, USA) was monitored using a probe taped on the hind limb to assure proper blood oxygenation (95–99%). The skin hair was clipped and cleaned with an iodine solution. A sagittal skin incision from behind both eyes to the occipital bone was made and the periosteum was gently detached from the underlying bone to allow visualization of bregma. Using stereotaxic coordinates (anterior posterior 3.5 mm, and lateral to 3.5 mm, in reference to bregma on the right side only) [49], a burr hole (diameter 1.5 mm) in the bone was perform using a stereotaxic drill, then a 5μL Hamilton syringe containing 0.25 μL of a solution of 0.025 UI of collagenase (Type IV) (Sigma-Aldrich, Oakville, ON, Canada) prepared in sterile saline was lowered 6 mm ventral to the dura matter. One animal died during surgery leaving 7 animals in the collagenase group**.** Control animals received an equal volume of sterile saline (0.9% NaCl) using the same methods. Injections were performed over 2 min then the needle was kept in place for an extra 5 min to allow good diffusion of the solution and prevent reflux along the needle track. The needle was then slowly withdrawn and the skin was closed using simple discontinuous Monocryl 4.0 sutures (Ethicon, Johnson & Johnson). The animals were singly caged to allow for a smooth recovery.

### Behavioral study

All animals were trained daily on all behavioral tests (rotarod, Hargreave, von Frey and acetone tests) for one week prior to the beginning of the study. Finally, mechanical allodynia was evaluated using Von Frey filaments. Baseline values were obtained 3 days prior to surgery. After surgery, animals were given one day to recover then were tested on days 2, 4, 8, 11, 15, 18, 22 post-surgery to evaluate if neuropathic pain was present and persisted over time. Tests were always performed in the morning to avoid circadian variations. Rats were acclimated for 15 min in the room prior to the beginning of testing.

### Rotarod test

To evaluate if the surgery and the treatment had any effect on motor coordination, animals were evaluated on the rotarod treadmill (Rotarod ENV-576, Med Associates Inc., St-Albans, VT, USA). The rotarod was set to the acceleration mode of 5 to 35 revolutions per min over 5 min. The maximum time the animal stayed on the rotarod (up to a maximum of 5 min), was recorded for each performance.

### Von Frey mechanical sensitivity test

All animals were evaluated with von Frey filaments (Stoelting, Wood Dale, IL, USA) as described by Chaplan et al. [50] to establish mechanical sensitivity. Rats were placed on a customized platform with mesh floor in Plexiglas chambers. Testing sessions began after 15 min habituation to the experimental setup. Testing was done by applying Von Frey filaments through the grid floor on the central region of the plantar surface of the rat’s hind paws, avoiding the foot pads. The filaments were applied only when the rats were stationary on 4 paws. A trial consisted of applications of the different filaments ranging from 4 to 22 g, starting with the smallest filament. Pressure was applied until buckling occurred for approximately 3 seconds. Rats were tested in groups of 4 animals and to avoid the effect of anticipation, a first paw was tested in all animals and then the opposite paw in the same fashion. The test began with either the right or the left hind paw alternatively. When no reaction was seen for a given filament, the next filament with higher rigidity was applied. If for a given filament, the animal withdrew its hind paw, the filament of lower rigidity was applied to evaluate the absence of withdrawal and then with the greater diameter filament to confirm the threshold. The force corresponding to the smallest filament causing a withdrawal reaction was recorded. The force corresponding to each filament was measured with the use of a weighing scale, at the beginning and at the end of the study to confirm force consistency.

### Hargreave thermal sensitivity test

Thermal sensitivity was evaluated using a Hargreaves apparatus (IITC Life Science, CA, USA) as previously described [51]. Each animal was placed in a Plexiglas chamber with the ground floor made of heated glass (27-31°C). Animals were allowed to acclimate to the experimental set up for 15 min prior to testing. Then radiant heat generated by a high intensity light bulb (40 W) was directed to the plantar surface of the hind paw. The lamp generated noxious heat stimulus. The time the animal took to lift its paw from the floor was recorded and noted as the thermal threshold. Rats were tested in groups of 4 animals and to avoid the effect of anticipation, a first paw was tested in all animals and then the opposite paw in the same fashion. The test began with either the right or the left hind paw alternatively to prevent any anticipatory behavior. A cut off time of the radiant stimulation was set at 20 sec to minimize tissue injury.

### Acetone test

This test was performed according to the method previously described by Choi et al. [52] With the animals standing on a mesh floor, acetone (25 μL) was applied to the plantar surface of the hind paw with a syringe without touching the paw. The number of movement of the paw and duration of the behavior were recorded for 30 s following the acetone application. Both hind paws were tested alternatively. . Responses to acetone were graded on the following scale: 0 = no reaction, 1 = mild reaction characterized by quick withdrawal (less than 3 movements) or short duration of lifting paw (less than 3 seconds), 2 = longer withdrawal or repeated movements (≥3 movements or ≥3 seconds). Animals considered truly reactive to acetone to determine the percentages of reactive animals are the ones graded 2. Data are presented in Figure 4 as the percentage of animal in each group that reacted to acetone.

### Treatment with ketamine

At the end of the behavioral study, animals were tested following the administration of ketamine. This short term evaluation was performed as a preliminary study, planned uniquely to be indicative of the potential therapeutic value of ketamine. Importantly, the operator was blinded to the treatments (type of drug and dose given) administered to the animals. The rats from the sham and the collagenase groups received IP injection of ketamine (100 mg/mL; Vetalar Bioniche Animal Health, Belleville, ON) at 1, 10 and 25 mg/kg on days 28, 30 and 32 post-surgery respectively. Behavioral tests started 30 min after the injection of the ketamine solution.

### Histological methods

Under deep isoflurane anesthesia, the abdominal cavity was opened and the abdominal aorta was clamped. Rats were perfused with an intracardiac infusion of first a physiological dextrose-sucrose solution (100 mL/rat; solution composition (1 L) : 8 g NaCl, 4 g dextrose, 8 g sucrose, 0,23 g calcium chloride), followed by a 10% buffered formalin solution (100 mL/rat). The brains were removed and fixed in formalin for a minimum of 48 h prior to being embedded in paraffin. Transverse sections (4 μm thick) from the thalamus were made using a microtome. The sections were stained with hematoxylin and eosin and cresyl violet using standard methods. Immunohistochemistry for glial fibrillary acidic protein (GFAP) was performed prior to microscopic evaluation. Polyclonal antibodies were obtained from BioGenex Laboratories (San Ramon, CA). The immunoglobulin fraction was composed of rabbit antisera diluted in PBS (pH 7.6) in 1% bovine serum albumin. Antibodies were stained with a commercially available immunoperoxidase procedure (Vectastain ABC kit, Vector Laboratories, Burlingame, CA). In addition, positive and negative controls for all brain slices were provided for proper identification of reactive astrocytes. Sections were reviewed by a board certified pathologist (Dr Pierre Hélie DVM, DACVP, Department of Veterinary Pathology, University of Montreal).

### Bioanalytical methods

At the end of the study (day 35), 6 rats (3/dose) were used to measure plasmatic concentrations of ketamine following an IP administration of 10 or 25 mg/kg. Under isoflurane anesthesia for a short period of time (less than 2 min), jugular blood samples (0.5 mL) were collected in sodium heparin tubes at 30 min, 1 and 2 h post injection. Blood samples were maintained on ice and centrifuged (3200 g for 10 min) within 30 min of collection. They were then stored at −80°C pending analysis by tandem liquid chromatography-mass spectrometry.

The analysis of ketamine was performed using a high performance liquid chromatography tandem mass spectrometer (HPLC-MS/MS). Briefly, the HPLC system consisted of a Perkin Elmer Series 200 (Boston, MA, USA) and a SCIEX API 2000 QTRAP hybrid MS system (AB Sciex, Concord, ON, Canada). Data acquisition and analysis were performed using analyst 1.5 (Concord, ON, Canada) and PRISM version 5.0 d) GraphPad software (La Jolla, CA, USA). Calibration curves were calculated from the equation y = ax + b, as determined by weighted (1/×) linear regression of the calibration line constructed from the peak-area ratios of the drug and the internal standard. Ketamine was extracted from rat plasma using a protein precipitation method. Fifty μL of each sample was mixed with 250 μL of internal standard solution (100 ng/mL of dextromethorphan in 50:50 acetone : methanol) in a 1.5 mL centrifuge tube. Samples were centrifuged at 12 000 g for 10 min and 200 μL of the supernatant was transferred into a 400 μL injection vial. The chromatographic separation was performed using an isocratic mobile phase with a Thermo Hypersil Phenyl 100 × 2 mm (5 μm) column. The mobile phase consisted of acetonitrile, methanol and 0.5% formic acid in water at a ratio of 60:20:20, respectively. The flow rate was fixed at 0.3 mL/min. Two μL of the extracted sample was injected and the total run time was set to 3 min. The mass spectrometer was interfaced with the HPLC system using a pneumatic assisted electrospray ion source. The Nitrogen gas 1 was set to 25 PSI, the Nitrogen gas 2 was set to 40 PSI and the electrospray electrode was set to 4000 V. The declustering (DP) potential was set to 15 V and the collision energy (CE) was set to 30 V. The selected reaction monitoring (SRM) transitions were set to m/z 238 → 163 and 272 → 215 for ketamine and dextromethorphan (internal standard) respectively. The dwell time was set to 150 msec and the pause time at 5 msec. For the bioanalytical method performance, a linear regression (weighted 1/concentration) was judged to produce the best fit for the concentration-detector relationship. The analytical ranges used were from 10 to 2 500 ng/mL. Observed coefficients of determination (R^2^) were ≥ 0.9913. The precision obtained ranged from 10.1% - 13.8% for and the accuracy observed was 97.0% - 104.3%. The limit of quantification was set at 10 ng/mL, according to the bioanalytical validation guideline published by the FDA, acceptable precision and accuracy results were achieved.

### Statistical analyses

The statistical analyses of behavioral data were conducted using a 2-way ANOVA with repeated measures and post hoc Tuckey tests performed with SAS (version 9.2, SAS Institute, Cary, NC, USA). For evaluation of cold allodynia, Cochran-Mantel-Haenszel test for ordinal data was used. For the ketamine behavioral results t-test were used to compare shams to controls. Results are presented as means and standard errors of the mean. Analyses revealing p values < 0.05 were considered statistically significant.

## Abbreviations

CPSP: Central post stroke pain; VPL: Ventral posterolatreal nucleus; IP: Intra-peritoneal; HPLC-MS: High performance liquid chromatography tandem mass spectrometer; NMDA: N-methyl-D-aspartate.

## Competing interests

The authors declare that they have no competing interest.

## Authors’ contributions

AC: animal experimentation, analysis and interpretation of the data, writing of manuscript. PH: histopathology, manuscript review. FB: analysis of ketamine by LC-MS/MS, manuscript review. PV: obtained funding, study design, supervised the experimentation, interpretation of the data, manuscript writing. All authors read the final version of the submitted manuscript.
